# Psychological health and motivational factors of health workers and community volunteers in preventive chemotherapy programs for neglected tropical diseases control in Ekiti State, Nigeria

**DOI:** 10.1186/s12889-025-25839-7

**Published:** 2025-12-01

**Authors:** Hammed O. Mogaji, Abiodun M. Lawal, Kayode H. Ojo, Aladejana Abdulrahman, Adebayo Boluwatife, Olowo Adejumoke, Fasakin Temidayo, Ismail Happiness, Fajana Oyinlola, Hilary I. Okoh, Francisca O. Olamiju, Uwem F. Ekpo

**Affiliations:** 1 Department of Animal and Environmental Biology, Parasitology and Epidemiology Unit, Federal University Oye-Ekiti, Oye-Ekiti, Ekiti State Nigeria; 2 Department of Psychology, Clinical Psychology Unit, Federal University Oye-Ekiti, Oye-Ekiti, Ekiti State Nigeria; 3 Neglected Tropical Diseases Unit, Ekiti State Primary Health Care and Development Agency, Ado-Ekiti, Ekiti State Nigeria; 4 Mission To Save The Helpless (MITOSATH), Jos, Nigeria; 5 Department of Pure and Applied Zoology, Parasitology and Epidemiology Unit, Federal University of Agriculture, Abeokuta, Ogun State Nigeria

**Keywords:** Stress, Anxiety, Depression, Health workers, NTDs, COVID-19, Nigeria

## Abstract

**Background:**

Mass Drug Administration (MDA) remains a key tool for elimination of Neglected Tropical Diseases (NTDs) and relies on the effort of health workers and community volunteers. Little is known about the mental health and motivation of these health workers to support better programming. This study assesses the psychological well-being of these implementers at two time periods (before and during the 2019 COVID-19 pandemic) to inform sustainable program strategies.

**Methods:**

We conducted a cross-sectional study in three pre-selected communities with a history of poor MDA performance in Ekiti State. We recruited 35 experienced implementers (health workers (*n* = 12), teachers (*n* = 11) and Community Drug Distributors (CDDs) (*n* = 12)) and assessed their psychological well-being using Perceived Stress Scale (PSS), Generalized Anxiety Disorder scale (GAD-7), and the Patient Health Questionnaire (PHQ-9), alongside motivational factors at intrinsic, societal, and organizational levels. Data were analyzed in R software, employing descriptive statistics, chi-square/Fisher’s exact tests, and Wilcoxon signed-rank tests to explore psychological and motivational changes before and during the pandemic.

**Results:**

Our findings show high self-reported levels of stress (86% vs. 94.3%, *p* > 0.05), anxiety (63% vs. 71%, *p* > 0.05), and depression (91.4% vs. 100%, *p* > 0.05) before and during the pandemic, respectively. Overall, CDDs were the most affected group, followed by health workers and teachers (*p* = 0.057). However, teachers showed a significant increase in stress (66.7% to 91.7%, *p* < 0.05) and anxiety (41.3% to 75%, *p* < 0.05) during the pandemic. Implementers were primarily motivated by community trust, opportunities to gain knowledge, help communities, and access to information. At the community level, admiration, support, recognition, and respect from friends and community members served as key motivators. However, organizational-level factors did not motivate health workers.

**Conclusion:**

The findings from this study highlight the need to promote psychosocial support through mental health resources and stress management to improve retention and effectiveness of MDA programs. Also, programs should explore how to tap into values such as community trust, support and recognition, alongside addressing organizational gaps to sustain participation and reduce burnout.

## Background

Neglected Tropical Diseases (NTDs) affect more than a billion people worldwide, with transmission closely tied to poor environmental conditions and human behaviors [1]. NTDs continue to impose a substantial global burden, with recent WHO estimates indicating approximately 119,000 deaths and 14.1 million disability-adjusted life years (DALYs) annually [2]. Beyond mortality and disability, NTDs contribute to reduced productivity, long-term incapacitation, stigmatization, and diminished socioeconomic and educational opportunities, affecting more than 1 billion people worldwide [2, 3]. These consequences impose a substantial financial burden on those affected and their families, trapping them in a state of economic disadvantage. The World Health Organization (WHO) has committed substantial resources to control and potentially eliminate NTDs in endemic regions using preventive chemotherapy (PC), with routine large-scale administration of safe, efficacious medications to populations considered too vulnerable in endemic settings, a strategy known as Mass Drug Administration (MDA) [4]. MDA implementation is tailored to endemicity levels, with coverage and timelines set by WHO guidelines to interrupt transmission [4]. In most endemic settings, MDA have prioritized five NTDs—onchocerciasis, lymphatic filariasis, trachoma, schistosomiasis, and soil-transmitted helminthiasis [4]. Notably, more than 40% of the one billion people at risk of NTDs reside in Africa and are affected by a combination of these priority NTDs [5]. This region has received over 2.7 billion treatment doses, with 19 of its 54 countries achieving the elimination of at least one NTD as at 2024 [2].

Elimination targets set for NTDs are reliant on successful MDA implementation, i.e., high coverage, or proportion of at-risk populations consistently swallowing the medicines over the prescribed timeframe [6]. This implementation framework relies heavily on efforts of indigenous community stakeholders, such as frontline health workers (nurses or community health workers), teachers and community volunteers—commonly referred to as Community Drug Distributors (CDDs)—who play a critical role in medicine distribution within the communities and schools [7]. However, MDA like all other public health programs is not immune to operational and implementation bottlenecks. MDA faces operational challenges, including urbanization [8, 9], migration, insecurity, and disruptions during pandemics like COVID-19 [10–14]. For instance, the 2019 COVID-19 pandemic caused a global pause in implementation through 2020 [11, 12], resulting in a substantial decline in the number of individuals treated from 1.21 billion in 2019 to 798 million in 2020. Although activities resumed in 2021, the recovery remains partial and far below pre-pandemic levels [13, 14].

Frameworks that monitor performance of MDA have been embedded within national programs to ensure high therapeutic coverage is achieved, and challenges hindering the achievement of elimination goals are addressed. Studies examining perception of MDA beneficiaries [12], MDA participation rates [14], and beneficiaries behaviors and mental health concerns [15] have highlighted factors such as persistent misconceptions about NTDs, fear of adverse drug reactions, social stigma, and logistical challenges that contribute to refusal and low participation rates. These insights have informed program adaptations, including more rigorous awareness campaigns and targeted messaging to dispel myths, improve trust, and increase uptake. Additional efforts, such as studies focused on migrant and mobile populations are helping to address context-specific drivers of low participation and improve demand for both MDA and surgical interventions aimed at reducing disease burden.

Despite this growing body of work on beneficiaries, the psychosocial toll on implementers (frontline health workers, CDDs and teachers) who drive MDA implementation remains underexplored. Existing reports acknowledge that these implementers often face heavy workloads, unpaid or underpaid labor, limited training, and community resistance [7, 16–18], which are more pronounced for CDDs and teachers, many of whom serve as volunteers without formal training or employment within the health workforce. We posit that such demands, beyond limiting their performance [17], may also contribute to stress, burnout, and broader mental health concerns, especially implementers working with populations affected by psychosocial or mental health conditions [19]. Collectively, these gaps point to an urgent need for interventions that prioritize implementers’ wellbeing, including support to manage stress, anxiety, and depression. Hence, in this study, we aim to assess the psychological health (stress, anxiety, and depression) and the intrinsic and extrinsic motivational factors of these implementers at two time-points (before and during the COVID-19 pandemic). By doing so, we aim to generate evidence that can inform the development of targeted support strategies that strengthen implementer wellbeing and enhance the sustainability and effectiveness of MDA.

## Methodology

### Ethical approval and consent to participate 

This study received ethical approval from the Ekiti State Ethical Review Board (MOH/EKHREC/EA/P/33). Formal permission was also obtained from the State NTD Control Unit. Participation in the study was entirely voluntary, and no respondent was coerced or financially induced to participate. Written informed consent was obtained from all participants after a clear explanation of the study purpose, procedures, potential risks, and benefits. Participants were informed of their right to withdraw at any time without penalty. Confidentiality was strictly maintained, and no identifying information was included in the data collection tools, and all responses were anonymized using unique participant codes. Completed questionnaires and electronic datasets were stored securely, with physical documents kept in locked cabinets accessible only to the research team. All study procedures adhered to the ethical principles of the Declaration of Helsinki (1964; most recently amended in 2008) and relevant national guidelines governing research involving human participants.

### Study area

This study was part of a larger study aimed at evaluating the impact of the COVID-19 pandemic on PC [14]. The aim was to inform the development and implementation of tailored strategies to enhance the effectiveness of MDA [14]. The research was conducted between May and July 2022 in Ekiti State, a region endemic for neglected tropical diseases (NTDs) in southwestern Nigeria. Ekiti comprises 16 Local Government Areas (LGAs), with its administrative capital situated in Ado-Ekiti.

### Study design and selection of participants

This study was cross-sectional in design involving administration of pre-tested questionnaires to collect quantitative data from a subset of implementers operating in three pre-selected communities: Ogbonjana, Oke-Osun, and Okekere. These communities were deliberately chosen due to their history of poor performance (i.e., low coverage of albendazole administration) during MDA conducted before the COVID-19 pandemic [13]. Given the small number of communities involved and the limited pool of healthcare workers engaged in community-level NTD programs, the sampling frame was intentionally narrow. The recruitment criteria required participants to have experience in at least three PC programs prior to 2020, regardless of age or gender. In each community, we aimed to recruit five implementers from three categories: frontline health workers (FLHFs), community-directed distributors (CDDs), and teachers, targeting 15 participants per community. However, we were able to recruit 11 CDDs, 12 FLHFs, and 12 teachers who met the eligibility requirements, along with one district NTD coordinator, resulting in a total of 35 participants. Of these, 19 participants had previously been involved in the larger survey as facilitators, while 16 implementers were added.

### Assessment of psychological status

We used pretested tools [20] to assess levels of stress, anxiety, and depression among health workers before and after the COVID-19 pandemic. These tools included three modules designed to measure distinct aspects of mental health: the Perceived Stress Scale (PSS), the Generalized Anxiety Disorder scale (GAD-7), and the Patient Health Questionnaire (PHQ-9). Each module offered a structured framework for evaluating the psychological well-being of the participants, with specific scoring systems for categorization. The PSS module comprised 10 questions focusing on participants’ feelings and thoughts, asking them to indicate the frequency of specific experiences. Responses were rated on a 5-point Likert scale ranging from “never” to “very often,” and each answer was assigned a score. Scores were summed to categorize stress levels as low (0–13), moderate (14–26), or high (27–40) [21]. The GAD-7 module evaluated anxiety through seven questions. Responses were scored on a 4-point Likert scale, ranging from “no anxiety” to “severe anxiety.” Total scores were used to classify anxiety levels as no anxiety (0–5), mild anxiety (5–9), moderate anxiety (10–14), or severe anxiety (>15) [22]. Lastly, the PHQ-9 module assessed depression levels through nine questions, with answers rated on a 4-point scale from “not at all” to “nearly every day.” Scores were classified as minimal depression (1–4), mild depression (5–9), moderate depression (10–14), moderately severe depression (15–19), or severe depression (20–27) [23]. The PSS, PHQ-9, GAD-7 tools used are attached as supplementary files and accessible at https://zenodo.org/records/14564157.

### Assessment of motivational factors

The motivation of health workers was assessed using an adapted tool based on published frameworks [17, 24]. The assessment tool considered key determinants of motivation, categorized into individual, societal/cultural, and organizational/structural factors. Each item was neutrally phrased as, “When you think about [factor], how motivated are you to perform MDA on a scale of 0–4?”. Responses were collected using a five-point Likert scale, where 0 indicated “not at all,” 1 corresponded to “a little bit motivated,” 2 to “moderately motivated,” 3 to “very motivated,” and 4 to “extremely motivated.”. The tool is attached as supplementary files and accessible at https://zenodo.org/records/14564157.

### Data management and statistical analysis

Data collected electronically using Kobocollect. However, data collected manually due to unforeseen circumstances were entered into computers by the research team and transmitted to a dedicated server. Data was subjected to statistical analysis in R software (v 4.3.2). Descriptive statistics such as frequencies, proportions, means, median and interquartile range (IQR) were used to summarize socio-demographic characteristics, stress, anxiety and depression level. Heat maps were used to show variations in responses among implementers. Pearson chi-square/Fisher’s exact test were used to test associations between categorical variables for stress, anxiety and depression scores. For the assessment of determinants of motivation in MDA programs, median and IQR, were estimated based on scores obtained from a four-point Likert scale. A median score equal to or greater than 3.0 (on a scale of 0–4) indicates that most respondents viewed the factor as a strong motivator for participation, while a score below 3.0 suggests a lack or weak motivation related to that factor. To examine changes in motivation before and during the pandemic, a Wilcoxon signed-rank test was performed on paired observations using the *wilcox.test* function. The significance level was set at 95%. Data analyzed and R scripts are attached as supplementary files and openly accessible at https://zenodo.org/records/14564157.

## Results

### Demography of study participants

Table 1 provides a demographic overview of the thirty-five implementers recruited across the three study communities: Ogbonjana (*n* = 8), Oke-Osun (*n* = 16), and Okekere (*n* = 11). By sex, more than 80% of implementers were female, had median ages between 40 and 50 years, and averagely with 5 to 6 years of experience years implementing MDA. By categories, implementers included front-line health workers, CDDs, and teachers. Front-line health workers comprised 50% of implementers in Ogbonjana, 40% in Oke-Osun, and 18.2% in Okekere. CDDs made up 37.5% in Ogbonjana, 33.3% in Oke-Osun, and 27.3% in Okekere. Teachers were most represented in Okekere at 54.5%, compared to 26.7% in Oke-Osun and 12.5% in Ogbonjana.

**Table 1:** Demographic characteristics of the study participants

	Communities		
	Ogbonjana	Oke-Osun	Okekere
	*N* = 8	*N* = 16	*N* = 11
Sex			
Male	1 (12.5)	3 (18.8)	2 (18.2)
Female	7 (87.5)	13 (81.2)	9 (81.8)
Age and years of experience			
Age in years*	40 (35, 43)	42 (34, 47)	50 (42, 50)
Year of PC Experience*	5.0 (4.3, 5.5)	5.0 (2.8, 10.0)	6.0 (4.0, 10.0)
Category			
Front-line Health Workers	4 (50)	6 (40)	2 (18.2)
CDDs	3 (37.5)	5 (33.3)	3 (27.3)
Teachers	1 (12.5)	5 (26.7)	6 (54.5)

*Median (IQR)

### Stress status of study participants

**Fig. 1:**
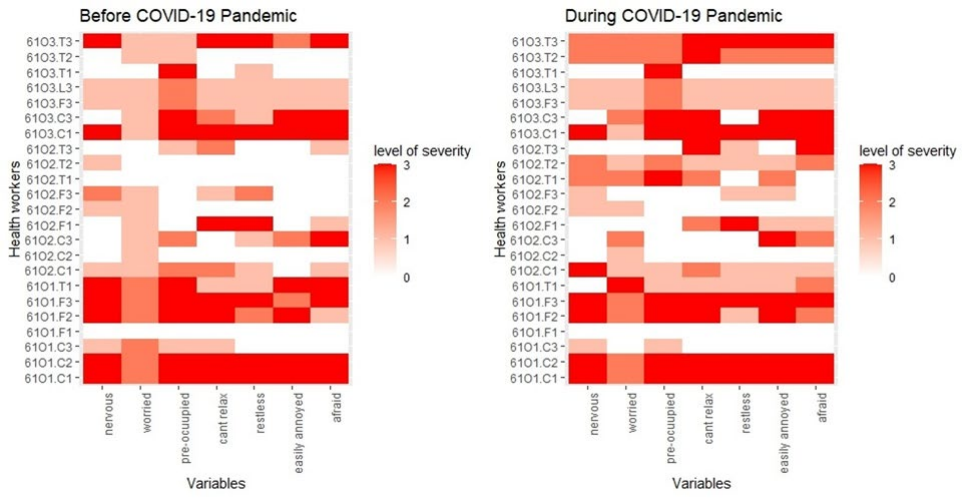
Heat Map showing Perceived Stress among study participants

Approximately 86% of them were moderately stressed before the pandemic, increasing to 94.3% during the pandemic, with no significant difference in proportions between the two periods (*p* = 0.23). Similarly, there were also no significant differences in stress status based on sex (*p* = 0.99) or age (*p* = 0.99). However, by community, implementers in Ogbonjana (100%) and Okekere (100%) were more stressed before the pandemic compared to those in Oke-Osun (68.8%) (*p* = 0.031). During the pandemic, stress levels across the communities showed no significant variation (*p* = 0.57). Additionally, stress levels varied marginally by categories of implementers before the pandemic. CDDs were the most stressed (100%), followed by frontline health workers (91.7%) and teachers (66.7%) (*p* = 0.057). During the pandemic, no significant variation in stress levels was observed across the categories (*p* = 0.57), but the severity of stress increased for teachers and health workers between the two periods, compared to CDDs with a slight reduction in proportion of those who had moderate stress. Figure 1 illustrates the heat map of stress scores for each variable in the Perceived Stress Scale, mapped against the identification codes of participants. The overall mean stress score was 18.5 (SD = 4.6) before the pandemic, which slightly decreased to 18.46 (SD = 3.54) during the pandemic. No significant changes in stress patterns were observed between the two periods (Table 2).

**Table 2:** Stress status of participants before and during the pandemic

		Stress Status					
	Number Recruited (35)	Before COVID-19			During COVID-19		
		Low	Moderate	*p*-value	Low	Moderate	*p*-value
Prevalence	35(100)	5(14.2%)	30(85.7)	-	2(5.7)	33(94.3)	0.23*
Sex							
Male	6(17.1)	1(16.7)	5(83.3)	0.99	0(0)	6(100)	0.99
Female	29(82.9)	4(13.8)	25(86.2)		2(6.9)	27(93.1)	
Age in years							
18–40	16(47.1)	2(12.5)	14(87.5)	0.99	1(6.2)	15(93.8)	0.99
> 40	18(52.9)	3(16.7)	15(83.3)		1(5.6)	17(94.4)	
Community							
Ogbonjanna	8(22.9)	0(0)	8(100)	0.031	0(0)	8(100)	0.70
Okeosun	16(45.7)	5(31.2)	11(68.8)		1(6.2)	15(93.8)	
Okekere	11(31.4)	0(0)	11(100)		1(9.1)	10(90.9)	
Implementer							
FLHW	12(34.3)	1(8.3)	11(91.7)	0.057	0(0)	12(100)	0.57
CDDs	11(31.4)	0(0)	11(100)		1(9.1)	10(90.9)	
Teachers	12(34.3)	4(33.3)	8(66.7)		1(8.3)	11(91.7)	

*FLHW* Frontline Health Worker

*p-value for comparison of the two time periods

### Anxiety status of study participants

**Fig. 2:**
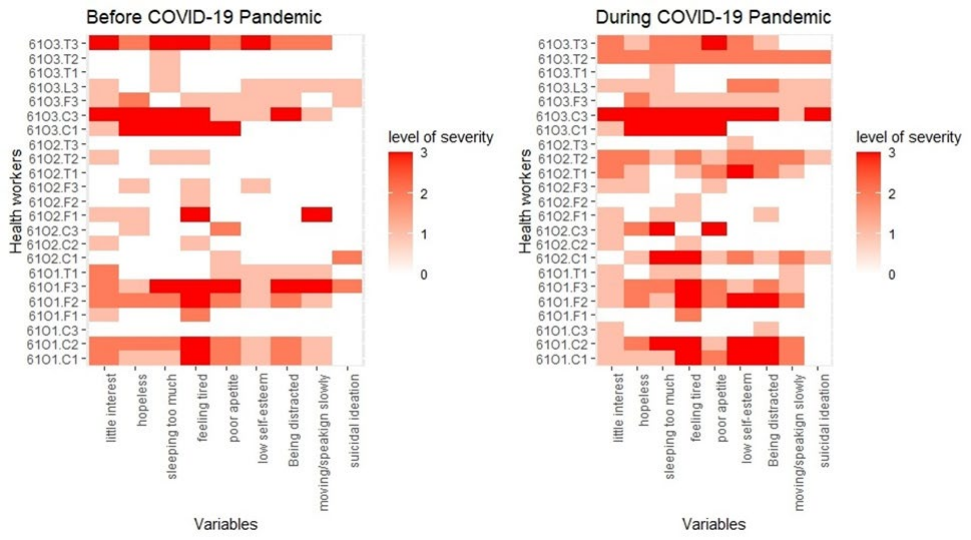
Heat Map showing Perceived Anxiety Status among study participants

About 63% of the participants experienced anxiety before the pandemic, which increased to 71% during the pandemic. Moderate and severe anxiety were reported by 5.7% and 25.7% of participants, respectively, before the pandemic, rising to 23% and 20% during the pandemic, with no significant difference in proportions between the two periods (*p* = 0.23). Similarly, no significant differences in stress levels were observed based on sex or age, either before or during the pandemic (*p* > 0.05). By community, implementers in Ogbonjana (87.5%) and Okekere (72.7%) reported anxiety levels before the pandemic compared to those in Oke-Osun (43.8%) (*p* = 0.048). However, during the pandemic, anxiety levels rose in Oke-Osun and Okekere to 62.5% and 81.8%, respectively, with no significant variation observed between the communities during this period (*p* = 0.57). Among implementer categories, CDDs were the most affected, with anxiety prevalence of 91% before and 82% during the pandemic. Frontline health workers reported a consistent prevalence of 58.2% across both periods, while teachers experienced an increase in anxiety prevalence from 41.3% before the pandemic to 75% during the pandemic. Interestingly, the severity of anxiety among teachers decreased during the pandemic (16.7%) compared to the pre-pandemic period (33.3%), whereas severe anxiety levels remained unchanged among other implementer categories. Figure 2 shows the heat map of anxiety scores for each variable in the Perceived Anxiety Scale, mapped against the identification codes of participants. The overall mean stress score was 8.4 (SD = 3.6) before the pandemic, which slightly increased to 9.0 (SD = 6.2) during the pandemic. Persistent severity was observed in community 1 (Ogbonjanna) across the two periods for all the variables on the scales compared to other communities (Table 3).

**Table 3:** Anxiety Status of study participants

			Anxiety Status									
	Number Recruited (35)			Before COVID-19					During COVID-19			
		Overall		Mild	Moderate	Severe	*p*-value	Overall	Mild	Moderate	Severe	*p*-value
Prevalence	35	22(62.9)		11(31.4)	2(5.7)	9(25.7)	-	25(71.4)	10(28.6)	8(22.9)	7(20)	0.23*
Sex												
Male	6(17.1)	5(83.3)		3(50)	0(0)	2(33.3)	0.53	4(66.6)	2(33.3)	1(16.7)	1(16.7)	0.96
Female	29(82.9)	17(58.6)		8(27.6)	2(6.9)	7(24.1)		21(72.4)	8(27.6)	7(24.1)	6(20.7)	
Age in years												
18–40	16(47.1)	10(62.5)		5(31.2)	1(6.2)	4(22.2)	0.94	14(87.5)	5(31.2)	5(27.8)	4(25.0)	0.89
> 40	18(52.9)	12(66.7)		6(33.3)	1(5.6)	5(31.2)		11(61.1)	5(27.8)	3(18.8)	3(16.7)	
Community												
Ogbonjanna	8(22.9)	7(87.5)		1(12.5)	1(12.5)	5(62.5)	0.048	6(75.0)	1(12.5)	1(12.5)	4(50.0)	0.13
Okeosun	16(45.7)	7(43.8)		6(37.5)	0(0)	1(6.2)		10(62.5)	5(31.2)	5(31.2)	0(0)	
Okekere	11(31.4)	8(72.7)		4(36.4)	1(9.1)	3(27.3)		9(81.8)	4(36.4)	2(18.2)	3(27.3)	
Implementer												
FLHW	12(34.3)	7(58.3)		4(33.3)	1(8.3)	2(16.7)	0.15	7(58.3)	4(33.3)	1(8.3)	2(16.7)	0.74
CDD	11(31.4)	10(90.9)		6(54.5)	1(9.1)	3(27.3)		9(81.8)	3(27.3)	3(27.3)	3(27.3)	
Teachers	12(34.3)	5 (41.6)		1(8.3)	0(0)	4(33.3)		9(75.0)	3(25.0)	4(33.3)	2(16.7)	

*FLHW* Frontline Health Worker

* *p*-value for comparison of the two time periods

### Depression status of study participants

**Fig. 3:**
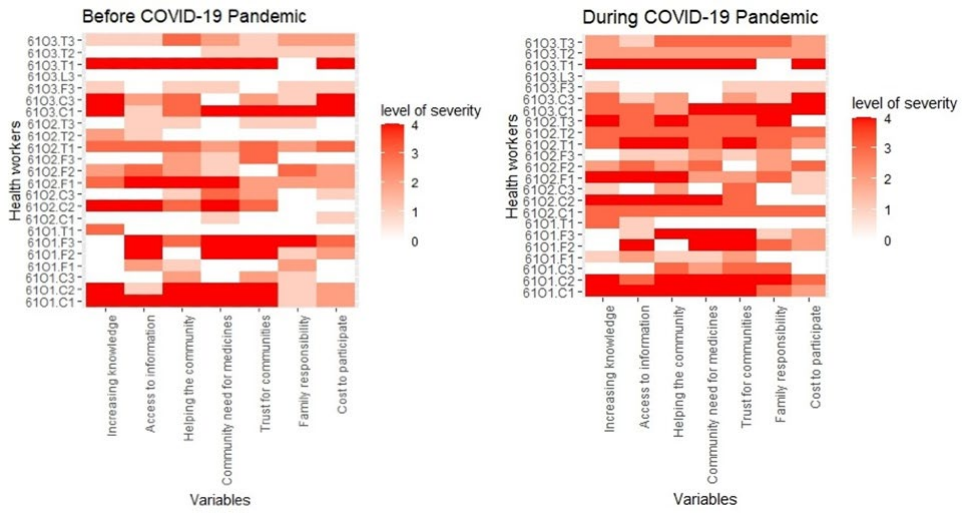
Perceived Depression Status among study participants

Overall, 91.4% of participants exhibited some form of depression before the pandemic, which increased to 100% during the pandemic (Table 4). Before the pandemic, 42.9% of participants had minimal depression, 25.7% mild depression, 5.7% moderate depression, and 8.6% each exhibited moderately severe and severe depression. During the pandemic, 40% of participants had minimal depression, 14.7% mild depression, 22.9% moderate depression, 17.1% moderately severe depression, and 5.7% severe depression. No significant differences in depression status were observed based on sex or age before or during the pandemic (*p* > 0.05). By community, all participants from Okekere (100%) exhibited some form of depression before the pandemic, compared to 87.5% in other communities, but the most severe form of depression was observed among participants in Ogbonjana. During the pandemic, all participants across the three communities experienced some form of depression, with increased severity in each community. For instance, in Okekere, severe depression rose from 9.1% to 18.2%; in Oke-Osun and Ogbonjana, moderately severe depression increased from 0% to 6.2%; and 25% to 50%, respectively. CDDs were the most affected during both periods, with a prevalence of 100% before and during the pandemic, compared to frontline health workers (FLHWs), with a prevalence of 91% and 100% before and during the pandemic, and teachers, with a prevalence of 83.3% before the pandemic and 100% afterward. The severity of depression remained constant (27.3%) among CDDs across both periods but increased among FLHWs from 8.3% to 16.7% and among teachers from 16.7% to 25%. Figure 3 shows the heat map of depression scores for each variable mapped against the identification codes of participants. The overall mean score was 7 (SD = 6) before the pandemic, which increased to 9.4 (SD = 7.1) during the pandemic. Severe depression was persistent in Okekere at both periods and emerged during the pandemic in the other two communities, notably for CDDs and FHLFs.

**Table 4:** Depression status of study participants

	Depression Status																
	Number Recruited (35)	Before COVID-19								During COVID-19							
		Overall	Minimal	Mild	Moderate	Moderately severe	Severe	*p*-value	Overall		Minimal	Mild	Moderate	Moderately severe		Severe	*p*-value
Prevalence	35	32(91.4)	15(42.9)	9(25.7)	2(5.7)	3(8.6)	3(8.6)	-	35(100)		14(40)	5(14.3)	8(22.9)	6(17.1)		2(5.7)	0.23*
Sex																	
Male	6(17.1)	6(100)	2(33.3)	2(33.3)	1(16.7)	0(0)	1(16.7)	0.60	6(100)		3(50)	1(16.7)	1(16.7)	1(16.7)		0(0)	0.95
Female	29(82.9)	26(89.7)	13(44.8)	7(24.1)	1(3.4)	3(10.3)	2(6.9)		27(93.1)		11(37.9)	4(13.8)	5(17.2)	5(17.2)		2(6.9)	
Age in years																	
18–40	16(47.1)	13(81.3)	6(37.5)	2(12.5)	1(6.2)	2(12.5)	2(12.5)	0.25	16(100)		6(37.5)	3(18.8)	4(25)	2(12.5)	1(6.2)		0.93
> 40	18(52.9)	18(100)	8(44.4)	7(38.9)	1(5.6)	1(5.6)	1(5.6)		17(94.4)		7(38.9)	2(11.1)	4(22.2)	4(22.2)		1(5.6)	
Community																	
Ogbonjanna	8(22.9)	7(87.5)	1(12.5)	2(25.0)	1(12.5)	2(25.0)	1(12.5)	0.14	8(100)		3(37.5)	0(0)	1(12.5)	4(50)		0(0)	0.088
Okeosun	16(45.7)	14(87.5)	11(68.8)	2(12.5)	0(0)	0(0)	1(6.2)		16(100)		8(50)	3(18.8)	4(25.0)	1(6.2)		0(0)	
Okekere	11(31.4)	11(100)	3(27.3)	5(45.5)	1(9.1)	1(9.1)	1(9.1)		11(100)		3(27.3)	2(18.2)	3(27.3)	1(9.1)		2(18.2)	
Implementer																	
FLHFs	12(34.3)	11(91.7)	6(50)	4(33.3)	0(0)	1(8.3)	0(0)	0.35	12(100)		6(50)	3(25)	1(8.3)	2(16.7)		0(0)	0.60
CDDs	11(31.4)	11(100)	4(36.4)	2(18.2)	2(18.2)	2(18.2)	1(9.1)		11(100)		3(27.3)	2(18.2)	3(27.3)	2(18.2)		1(9.1)	
Teachers	12(34.3)	10(83.3)	5(41.7)	3(25.0)	0(0)	0(0)	2(16.7)		12(100)		5(41.7)	0(0)	4(33.3)	2(16.7)		1(8.3)	

*FLHW *Frontline Health Worker

*p-value for comparison of the two time periods

### Individual-level determinants of motivation to participate in MDA programs

Table 5 shows the individual-level determinants of motivation among health workers before and during the COVID-19 pandemic. In Ogbonjana, trust for the communities and opportunities to have access to information were the strongest motivators for participating in MDAs before the pandemic, with a median score of 4.00 (IQR: 1.50, 4.00) and 3.00 (IQR: 0.75, 4.00), respectively. These motivations remained stable during the pandemic, with no significant changes observed in their median scores. In Oke-Osun, none of the factors strongly motivated participation in MDAs before the pandemic, as all determinants had a median score below 2.0. However, during the pandemic, significant improvements were noted in motivation for participation due to opportunities for access to information (median: 3.00, IQR: 1.00, 4.00; *p* = 0.04), and opportunities to help the community (median: 3.00, IQR: 2.00, 4.00; *p* = 0.03). In Okekere, opportunities to help the community emerged as the only strong motivator before the pandemic, with a median score of 3.00 (IQR: 1.00, 3.50). During the pandemic, motivation increased due to trust for the communities (median: 3.00, IQR: 2.00, 4.00; *p* = 0.05) and opportunities for increasing knowledge (median: 3.00, IQR: 2.00, 3.50). Community pressure for medicines also contributed to motivation (median:3.00, IQR:1.00, 4.00), though changes were not statistically significant.

**Table 5:** Individual-level determinants of motivation to participate in MDA programs

	Ogbonjanna (*N* = 8)			Oke-Osun (*N* = 16)			Okekere (*N* = 11)		
Individual-level determinants	Before COVID Median (IQR)	During COVID Median (IQR)	*p*-value	Before COVID Median (IQR)	During COVID Median (IQR)	*p*-value	Before COVID Median (IQR)	During COVID Median (IQR)	*p*-value
Opportunities for increasing knowledge	1.50 (0.00, 4.00)	2.00 (0.00, 3.25)	0.99	1.50 (0.00, 3.00)	2.50 (1.00, 3.25)	0.01	2.00 (1.00, 4.00)	3.00 (2.00, 3.50)	0.28
Opportunities to have access to information	3.00 (0.75, 4.00)	2.50 (1.00, 4.00)	0.99	1.50 (0.00, 3.25)	3.00 (1.00, 4.00)	0.04	1.00 (0.50, 3.00)	2.00 (1.00, 3.50)	0.17
Opportunity to help the community	2.50 (0.75, 4.00)	3.50 (0.75, 4.00)	0.35	1.50 (1.00, 3.00)	3.00 (2.00, 4.00)	0.03	3.00 (1.00, 3.50)	2.00 (1.50, 4.00)	0.78
Community pressure for the medicines	2.00 (0.00, 4.00)	3.00 (0.75, 4.00)	0.37	2.00 (1.00, 3.25)	3.00 (2.00, 3.00)	0.22	2.00 (1.00, 4.00)	3.00 (1.00, 4.00)	0.23
Trust for the communities	4.00 (1.50, 4.00)	4.00 (2.75, 4.00)	0.37	2.00 (1.00, 3.00)	3.00 (1.75, 3.00)	0.15	2.00 (1.00, 4.00)	3.00 (2.00, 4.00)	0.05
Family responsibilities	1.00 (1.00, 2.50)	3.00 (0.75, 3.25)	0.67	0.00 (0.00, 1.25)	2.00 (0.00, 3.00)	0.01	1.00 (0.50, 2.50)	2.00 (0.50, 3.50)	0.07
Relative cost to participate*	1.00 (0.00, 2.00)	1.00 (0.00, 2.00)	0.99	1.00 (0.00, 2.00)	1.00 (0.00, 3.00)	0.43	2.00 (1.00, 4.00)	2.00 (1.50, 4.00)	0.15

*N: Number of implementers recruited*

*IQR: Inter Quartile Range*

*Responses were on a five-point Likert’s scale*,* in which 0 corresponded to “not at all”*,* 1 to “a little bit motivated”*,* 2 to “moderately motivated”*,* 3 to “very motivated” and 4 to “extremely motivated”*

*Amount to be made while doing other jobs,* or amount to be saved if not doing MDA*

### Societal or Cultural-level determinants of motivation to participate in MDA programs

Across all the communities, none of the societal or cultural determinants motivated health workers to participate in MDA before the pandemic, with median scores below 2.0 (Table 6). However, during the pandemic, certain factors led to improvements in motivation. In Ogbonjanna, significant improvements were noted in motivation due to admiration from friends and community (median: 4.00, IQR: 1.50, 4.00), respect from friends and community (median: 4.00, IQR: 0.00, 4.00) and adverse reactions after MDA (median: 3.00, IQR: 1.50, 4.00) were also noted to motivate health workers, but no significant change was observed (*p* = 0.41, *P* = 0.42 and *p* = 0.41, respectively). In Oke-Osun, none of the societal or cultural determinants motivated health workers to participate in MDA before and during the pandemic. Similarly, in Okekere, none of the societal or cultural determinants motivated health workers to participate in MDA before the pandemic, however significant improvements in motivation were observed during the pandemic due to encouragement (median: 3.00, IQR: 1.50, 4.00; *p* = 0.05), support (median: 3.00, IQR: 2.50, 4.00; *p* = 0.09), recognition (median: 3.00, IQR: 3.00, 4.00; *p* = 0.07), respect (median: 3.00, IQR: 3.00, 4.00; *p* = 0.07), admiration from friends and community (median: 3.00, IQR: 2.50, 4.00; *p* = 0.18). Physical safety and security also showed a positive trend (median: 3.00, IQR: 1.00, 4.00, *p* = 0.09).

**Table 6:** Societal or Cultural-level determinants of motivation to participate in MDA programs

	Ogbonjanna (*N* = 8)			Oke-Osun (*N* = 16)			Okekere (*N* = 11)		
Societal/Cultural-level determinants	Before COVID Median (IQR)	During COVID Median (IQR)	*p*-value	Before COVID Median (IQR)	During COVID Median (IQR)	*p*-value	Before COVID Median (IQR)	During COVID Median (IQR)	*p*-value
COVID-19 pandemic	0.50 (0.00, 1.25)	1.00 (0.75, 1.25)	0.85	1.00 (0.00, 1.25)	2.00 (1.00, 3.00)	0.02	1.00 (0.00, 1.50)	2.00 (0.00, 2.00)	0.58
Appreciation from friends and community	1.00 (1.00, 1.25)	1.50 (0.75, 3.00)	0.57	2.00 (0.75, 2.00)	2.00 (1.00, 3.00)	0.22	1.00 (0.00, 2.00)	2.00 (1.00, 3.50)	0.06
Encouragement from friends and community	1.00 (0.75, 1.00)	2.00 (1.50, 2.50)	0.03	1.50 (0.75, 3.00)	2.50 (2.00, 3.00)	0.09	1.00 (1.00, 4.00)	3.00 (1.50, 4.00)	0.05
Support from friends and community	1.00 (0.00, 1.00)	1.00 (0.75, 1.50)	0.99	1.00 (0.00, 3.00)	2.50 (1.00, 3.00)	0.09	2.00 (1.00, 4.00)	3.00 (2.50, 4.00)	0.09
Recognition from friends and community	1.00 (0.00, 1.00)	1.00 (0.00, 3.25)	0.41	1.00 (0.00, 2.00)	1.50 (0.00, 2.00)	0.73	1.00 (1.00, 4.00)	3.00 (3.00, 4.00)	0.07
Respect from friends and community	1.50 (0.75, 4.00)	4.00 (0.00, 4.00)	0.42	1.00 (0.00, 2.00)	2.00 (0.00, 3.00)	0.22	1.00 (1.00, 4.00)	3.00 (3.00, 4.00)	0.07
Admiration from friends and community	2.00 (0.00, 4.00)	4.00 (1.50, 4.00)	0.17	1.00 (0.00, 2.00)	2.50 (1.00, 3.00)	0.01	2.00 (1.00, 4.00)	3.00 (2.50, 4.00)	0.18
Physical safety and security	1.00 (0.75, 1.00)	2.50 (0.00, 3.25)	0.09	0.50 (0.00, 2.25)	2.00 (1.50, 2.25)	0.08	1.00 (0.00, 4.00)	3.00 (1.00, 4.00)	0.09
Complaints from community	1.00 (1.00, 2.50)	2.00 (0.75, 2.00)	0.68	0.50 (0.00, 3.25)	2.00 (0.75, 3.00)	0.17	1.00 (0.00, 1.00)	1.00 (1.00, 2.50)	0.05
Disrespect from community	0.00 (0.00, 1.00)	2.00 (0.75, 2.25)	0.05	1.00 (0.00, 1.25)	2.00 (0.00, 3.00)	0.04	0.00 (0.00, 1.00)	1.00 (0.00, 2.50)	0.17
Adverse reactions after MDA	2.00 (0.75, 4.00)	3.00 (1.50, 4.00)	0.41	1.00 (0.75, 1.00)	1.50 (0.75, 3.00)	0.04	1.00 (0.00, 1.00)	1.00 (0.00, 2.50)	0.09

N: Number of implementers recruited

IQR: Inter Quartile Range

Responses were on a five-point Likert’s scale, in which 0 corresponded to “not at all”, 1 to “a little bit motivated”, 2 to “moderately motivated”, 3 to “very motivated” and 4 to “extremely motivated”

### Organizational-level determinants of motivation to participate in MDA programs

Table 7 summarizes the organizational-level determinants of motivation among health workers before and during the pandemic. Among the 15 factors assessed, only three—support from colleagues and supervisors, monetary and material incentives—were identified as positive motivators of participation in the MDA program. In Ogbonjana, support from colleagues and supervisors (Median: 3.00, IQR: 0.75, 4.00), as well as monetary incentives (Median: 4.00, IQR: 0.75, 4.00) and material incentives (Median: 4.00, IQR: 1.50, 4.00), were consistently strong motivators before and during the pandemic. In Oke-Osun, none of the organizational factors yield substantial motivation (Median scores were below 2.0). In Okekere, support from colleagues and supervisors remained a consistent motivator (Median: 3.00, IQR: 1.00, 4.00) before and during the pandemic.

**Table 7:** Organizational -level determinants of motivation to participate in MDA programs

	Ogbonjanna (*N* = 8)			Oke-Osun (*N* = 16)			Okekere (*N* = 11)		
Organizational-level determinants	Before COVID Median (IQR)	During COVID Median (IQR)	*p*-value	Before COVID Median (IQR)	During COVID Median (IQR)	*p*-value	Before COVID Median (IQR)	During COVID Median (IQR)	*p*-value
Support from colleagues and supervisors	3.00 (0.75, 4.00)	3.00 (0.00, 4.00)	0.99	0.00 (0.00, 1.25)	1.00 (0.00, 2.25)	0.03	3.00 (1.00, 4.00)	3.00 (1.00, 4.00)	0.99
Respect from supervisors	2.00 (1.75, 3.25)	2.50 (0.00, 4.00)	0.89	0.00 (0.00, 1.25)	1.00 (0.75, 2.00)	0.01	1.00 (0.00, 1.00)	1.00 (0.00, 1.50)	0.27
Complaints from supervisors	0.00 (0.00, 1.00)	1.00 (0.00, 1.25)	0.15	0.00 (0.00, 2.00)	0.50 (0.00, 2.00)	0.41	1.00 (0.00, 1.00)	1.00 (0.00, 2.50)	0.39
Transformative leadership	1.50 (0.00, 3.25)	2.00 (0.00, 4.00)	0.99	0.00 (0.00, 0.25)	1.00 (0.00, 1.00)	0.05	1.00 (0.50, 3.50)	2.00 (0.50, 4.00)	0.58
Monetary incentives	4.00 (0.75, 4.00)	3.50 (2.25, 4.00)	0.99	0.00 (0.00, 0.00)	0.00 (0.00, 1.00)	0.07	1.00 (0.00, 1.00)	1.00 (0.00, 2.00)	0.41
Material incentives	4.00 (1.50, 4.00)	3.50 (1.50, 4.00)	0.99	1.00 (0.00, 1.25)	1.00 (0.00, 2.00)	0.20	0.00 (0.00, 1.00)	1.00 (0.00, 2.00)	0.10
Supply of medicines	1.00 (0.00, 2.25)	1.00 (0.00, 2.25)	0.99	1.00 (0.00, 1.25)	1.00 (0.00, 2.00)	0.29	0.00 (0.00, 1.00)	1.00 (0.00, 2.00)	0.10
Storage of medicines	0.50 (0.00, 2.25)	0.50 (0.00, 3.00)	0.77	0.50 (0.00, 1.00)	1.00 (0.00, 2.00)	0.12	0.00 (0.00, 1.00)	1.00 (0.00, 2.50)	0.10
Training activities	1.00 (0.00, 4.00)	1.50 (0.00, 2.25)	0.79	0.00 (0.00, 1.00)	1.00 (0.75, 2.00)	0.01	0.00 (0.00, 1.50)	2.00 (0.50, 3.00)	0.10
Availability of materials and resources^a^	1.00 (0.75, 3.25)	2.50 (0.75, 4.00)	0.37	1.00 (1.00, 3.00)	2.00 (1.75, 3.00)	0.12	1.00 (0.00, 1.00)	1.00 (0.00, 2.50)	0.09
Workload^b^	1.00 (0.00, 1.00)	0.50 (0.00, 1.25)	0.77	0.50 (0.00, 1.00)	1.00 (0.75, 2.00)	0.03	1.00 (0.00, 2.00)	1.00 (1.00, 2.00)	0.37
Other training opportunities	0.00 (0.00, 1.25)	1.00 (0.75, 2.50)	0.27	1.00 (1.00, 3.00)	2.00 (1.00, 3.25)	0.44	1.00 (1.00, 3.50)	2.00 (1.00, 3.50)	0.37
Job security	1.50 (0.00, 2.00)	2.00 (1.75, 4.00)	**0.05**	1.00 (0.00, 2.00)	2.00 (1.00, 2.00)	0.15	2.00 (0.50, 3.50)	2.00 (0.50, 3.50)	1.00
Transportation	0.00 (0.00, 0.00)	0.00 (0.00, 1.25)	0.71	1.00 (0.00, 2.00)	1.00 (0.00, 2.00)	0.80	1.00 (0.00, 2.00)	1.00 (0.00, 3.50)	0.27
Non-availability of drugs to treat adverse events	0.50 (0.00, 1.00)	0.00 (0.00, 0.00)	0.09	0.00 (0.00, 1.00)	0.00 (0.00, 0.00)	0.23	0.00 (0.00, 1.00)	1.00 (0.00, 2.00)	0.35

*N: Number of implementers recruited*

*IQR: Inter Quartile Range*

*Responses were on a five-point Likert’s scale*,* in which 0 corresponded to “not at all”*,* 1 to “a little bit motivated”*,* 2 to “moderately motivated”*,* 3 to “very motivated” and 4 to “extremely motivated”*

^*a*^*Office space*,* dose poles and registers*

^*b*^ *Number of allocated households is too much*

## Discussion

The connections between NTDs and mental health have gained recognition over the years [25] with person-centered approaches towards improving quality of impact among those with clinical morbidities [26, 27]. Till date, little attention has been drawn to health workers and volunteers involved in MDA programs, as regards their mental health, and how this might have impacted their participation and work in such programs [28, 29]. Studies focusing on this group have always prioritized understanding general perception [30], challenges in PC implementation, and how to address them [31]. Here, perhaps for the first time, we provide information on psychosocial health and motivation of health workers for participating in MDA programs.

Mental health issues among healthcare workers are not uncommon and, in many cases, are expected. However, our findings reveal high levels of stress, depression, and anxiety, surpassing those reported in previous studies [28, 31–33]. This may represent the effect of combined responsibilities of serving on other health programs such as MDA, as we have hypothesized, or rather other wellbeing or societal issues, and warrants investigation. Furthermore, this study did not assess quality of life or investigate specific stressors in depth, the consistently elevated scores across both time points suggest a continuous source or mediator of depressed mental health rather than effects limited solely to unique pandemic-related experiences [34]. This interpretation is inferential, as our cross-sectional design cannot confirm temporal causality. Future longitudinal studies are therefore needed to test whether these observed mental health challenges are persistent and to identify the factors that mediate them. As with other studies, high stress has been implicated in a range of physiological (irritability) and psychological (low levels of self-confidence, poor concentration or coping mechanisms, and anger) consequences expressed by respondents in our study [35, 36], and could be a precursor to depression and anxiety [37]. Participants who reported high level of stress also had depressive symptoms such as excessive sleep, fatigue, low self-esteem, difficulty concentrating, and frequent distraction [38, 39]. Further to this, we observed that mental health states were not statistically related to gender and age [40], however, some trends (though insignificant changes between groups) were observed across the category of workers (health workers, CDDs and teachers) and time-period (before and during the pandemic). Notably, CDDs exhibited poorer mental health than healthcare workers, followed by teachers in relation to perceived workload or demand [41]. This finding aligns with expectations, as CDDs typically carry the heaviest workload [17]. Their responsibilities often include door-to-door drug distribution within a short time frame, frequently under physically demanding conditions that require extensive walking and repeated visits, sometimes in adverse weather. In contrast, teachers primarily conduct MDA activities within school settings, where medication administration takes place in classrooms and requires far less mobility than the community-wide distribution undertaken by CDDs. In addition, existing concerns about the remuneration model for CDDs’ work have contributed to attrition [42] and may serve as an additional mediator of stress. Among the three categories of implementers, CDDs constitute the only formal volunteer group—often comprising students, retired civil servants, artisans, or peasant farmers—who are not salaried through the health or education systems. Unlike health workers and teachers, whose regular employment provides stable income and for whom MDA incentives are supplementary, CDDs therefore may be more reliant on modest program allowances, making them more vulnerable to financial strain and workload-related stress. Furthermore, in two of the three communities studied, teachers experienced higher stress and anxiety levels during the pandemic. compared to other implementer groups. This pattern is consistent with findings from a systematic review of studies conducted in China, Brazil, the United States, India, and Spain, which reported elevated psychological distress among teachers during COVID-19 [43]. These increases have been attributed to school closures, the sudden shift to remote teaching, and the added responsibility of supporting students and their families [43]. In our setting, however, we posit that the heightened stress among teachers may also be linked to the financial impact of school closures. With reduced or disrupted income during this period, many teachers may have been compelled to take on additional jobs to sustain their livelihoods, thereby adding to their psychological burden [43, 44]. Addressing psychological well-being through stress management workshops, peer support groups, and counseling services can mitigate mental health challenges. Integrating regular wellness check-ins and mental health first aid training into program structure can provide much-needed support.

Beyond mental health challenges, understanding what drives implementers participation is critical for MDA sustainability. Foremost, at the individual level, we found community trust [17, 45], opportunities to gain knowledge [17, 46], help communities, and access information emerged as strong intrinsic motivators. This is at the expense of other weak motivators such as family responsibilities and amount to be made while doing other jobs. These observations are not new and align with other reports highlighting altruism, social recognition and improved knowledge as strong motivators [47, 48]. More importantly, the consistency of these motivators has been established in a systematic review done by Krentel et al. [18]. Similarly, at a societal level, participants reported feeling more motivated when they received admiration, support, recognition, and respect from friends and community members during the pandemic. While we did not quantify this shift, we infer that such recognition could have emerged earlier but only became more visible when the pandemic highlighted the importance of their roles. Nevertheless, respondents were generally less motivated by organizational or institutional factors, except for the Ogbonjana community, where motivation levels were comparatively higher. In this community, implementers were particularly motivated by monetary and material incentives. Although our study did not explore the underlying reasons in depth, a reasonable interpretation could be that Ogbonjana may have benefitted from access to additional funding or programmatic resources and a more supportive work environment as the Median and IQR were also higher for support from colleagues and supervisors. We believe such favorable working conditions may reinforce motivation and enhance willingness to participate in MDA activities, highlighting the importance of context-specific institutional support in sustaining engagement. Overall, our study revealed that implementers were not motivated to participate in MDA programs based on organizational-level determinants. For instance, CDDs—who are often students, retired civil servants, artisans, or peasant farmers—carry the heaviest workload and are the only implementer group that is not salaried [38]. Consequently, even when CDDs and health workers derive motivation from intrinsic factors or societal expectations, the lack of adequate organizational support can undermine these gains. This institutional neglect not only diminishes motivation but may also weaken program performance and sustainability.

Given the consistency of these motivational factors (community trust, admiration, support, recognition, and respect from friends and community members) across various NTD studies [17], it is surprising that they remain largely untapped in efforts to improve program performance. To enhance worker retention, it is crucial for programs to actively reinforce these values through practical initiatives within MDA programs. Pilot ideas that could be tested include those that favor recognition [49]. For instance, organizing periodic ceremonies to celebrate and reward volunteers for their dedication, particularly on significant days such as World NTD Day. Recognizing outstanding contributions with certificates, plaques, or small incentives—such as scholarships or professional training opportunities—can help volunteers feel valued and appreciated. Additionally, encouraging communities to participate in “Volunteer Appreciation Days” where members express gratitude through testimonials and public recognition can enhance social trust and motivation. These events could also feature storytelling sessions, allowing volunteers to share their experiences and the impact of their work.

### Limitation of this study

The findings of this study may be influenced by the limited participant size, a common challenge in most MDA programs, as only a small subset of the broader health workforce is involved. Future studies should consider recruiting implementers across more communities to enhance representativeness. Additionally, since the psychological health of implementers may be affected by external stressors, a more comprehensive demographic profiling—considering additional responsibilities and perceived levels of stress, anxiety, and depression based on those responsibilities—would provide valuable insights for developing targeted support programs. Lastly, we relied on self-reported perceptions, including recall-based assessments of participants’ motivation and psychological wellbeing before and during the pandemic. Such retrospective reporting introduces the possibility of recall bias, as participants may inaccurately remember or reinterpret their experiences over time. These factors may affect the accuracy of comparisons across the two recalled time points, and the findings should therefore be interpreted with caution.

## Conclusion

This study highlights the significant mental health challenges faced by health workers and volunteers involved in MDA programs, underscoring the need for greater attention to their psychosocial well-being. Despite high levels of stress, anxiety, and depression, intrinsic and societal motivators such as community trust, knowledge acquisition, opportunity to help others, alongside societal admiration, respect and recognition, were strong drivers of participation. However, organizational deficiencies, including inadequate support, poor remuneration models, and heavy workloads, undermine these gains and could lead to attrition. Addressing these gaps through enhanced organizational support and sustainable motivation strategies is critical to ensuring the retention and effectiveness of health workers in NTD programs.

## Data Availability

The datasets used and/or analyzed during the current study are openly accessible at [https://zenodo.org/records/14564157].
